# The physiotherapy workforce in the Brazilian Unified Health Care System

**DOI:** 10.1186/s12960-021-00642-8

**Published:** 2021-08-21

**Authors:** Carolina Hart Rodés, João Vitor Lovato Daré, Bruna Carolina de Araujo, Leonardo Graciani, Silvia Maria Amado João, Ana Claudia Camargo Gonçalves Germani, Ana Carolina Basso Schmitt

**Affiliations:** 1 Departamento de Fisioterapia, Fonoaudiologia e Terapia Ocupacional da Faculdade de Medicina, Universidade de São Paulo, São Paulo, SP Brazil; 2 Departamento de Geografia da Faculdade de Filosofia, Letras e Ciências Humanas, Universidade de São Paulo, São Paulo, SP Brazil; 3 Departamento de Medicina Preventiva da Faculdade de Medicina, Universidade de São Paulo, São Paulo, SP Brazil

**Keywords:** Physiotherapists, Health workforce/statistics & numerical data, Time factors

## Abstract

**Background:**

Maintaining sufficient health care workforce is a global priority to achieve universal health coverage. Therefore this study addresses the availability of physiotherapists in Brazil.

**Objective:**

To describe secular trends of the physiotherapy workforce-to-population ratio in the Unified Health System, considering public and private sector and care level (primary, secondary, tertiary) in Brazil and its regions.

**Method:**

Descriptive exploratory quantitative study based on secondary sources. All data related to the distribution of physiotherapists between August 2007 and September 2016 regarding facilities types, location and public and private sectors was obtained from the Brazilian National Registry of Health Care Facilities. Data related to the population of Brazil was extracted from Brazilian Institute of Geography and Statistics. The physiotherapy workforce-to-population ratio was calculated by the number of physiotherapists per 1000 population (public and private sector and care level) by ANOVA test. The distribution trends are represented on maps. Annual growth rates were estimated with Prais–Winsten linear regression models, with a significance level of 0.05, autocorrelation was checked by the Durbin–Watson test.

**Results:**

The physiotherapists ratio in Brazil was 0.22/1000 population in 2007 and 0.41 in 2016, showing growth of 86%, with an increasing trend of 0.5% on an annual average. The public sector had the biggest physiotherapy workforce in the country in 2007 and 2016. The primary health care had the smallest physiotherapy workforce-to-population ratio (2007: *p* > *0.001* and 2016: *p* = *0.003*), even though it had the largest growth trend in annual average (0.9% *p* > *0.001*), followed by public and private tertiary health care sectors (0.8% *p* > *0.001*). The workforce in secondary health care was bigger in the private sector than in the public sector (0.6% *p* > *0.001* vs. 0.2% *p* = *0.004*). Overall, all regions had greater growth of physiotherapy workforce-to-population ratio in public primary and tertiary health care sectors, and private secondary health care sector, mainly the Southeast, South and Central-West regions.

**Conclusion:**

Although the physiotherapy workforce in Brazil is relatively small, there was a trend towards growth with differences among care levels, and public and private sectors. The physiotherapy workforce-to-population ratio is bigger in the private secondary health care sector, followed by public tertiary, secondary and primary health care sectors. Sub-national regions show similar trends to the national estimates, with minor variations by region.

## Background

In order to identify actions that will promote advances in global health, the World Health Organization (WHO) periodically updates epidemiological data related to the healthcare workforce of partner countries. At the Third Global Forum on Human Resources for Health, an analysis of the WHO Global Health Observatory Data Repository containing information from 36 countries showed that maintaining a sufficient health care workforce is a global priority and that the effectiveness of that workforce should be determined by calculating the healthcare workforce-to-population ratio [1]. At the Fourth Global Forum on Human Resources for Health, four years later, besides acknowledging the urge to increase the recruitment and development of the health workforce, especially in developing countries, it was once more emphasized the fundamental importance of an optimally organized and distributed health workforce [2].

Despite the recommendation regarding universal health systems, considering health workers’ availability, accessibility, acceptability and quality to assure effective coverage [3], WHO has identified only five specific occupations (doctors, nurses, midwives, dentists and pharmacists) as indicators of the Sustainable Development Goals’ Target related to health workforce. That partly explains why most published literature about the health workforce ratio and its distribution only consider doctors [4–6] or doctors, nurses and midwives [7, 8].

In contrast, it is known that “one in every three people in the world would benefit from rehabilitation at some point during the course of their illness”, and most of them due to musculoskeletal disorders [9]. Despite the critical demand for rehabilitation workforce due to the growing burden of disability related to chronic conditions [9], there are no recommendations from the WHO regarding the ideal physiotherapy workforce-to-population ratio, nor regarding the criteria for activities and services related to health promotion, protection, recovery [3] and palliative care. The discussion about physiotherapy density has been included in the rehabilitation health workforce [10–12], and also in studies involving specifically the physiotherapy workforce-to-population ratio in different countries [13–16].

In Brazil, the Unified Health System (*Sistema Único de Saúde*—SUS) comprises both public and private sectors, and has been progressively evolving since 1988 (over 30 years), delivering universal and comprehensive health care to the Brazilian population. Different kinds of health professionals, including physiotherapists, have to work together in an integrated network to offer different services considering the three care levels: primary, secondary, and tertiary [17]. In all three care levels, physiotherapists skills have an important role in rehabilitation, while also embracing health promotion and injury prevention, contributing to a more comprehensive care [18].

Costa et al. [19] identified the distribution of physiotherapists among the various types of healthcare facilities in Brazil, but there is no data concerning the time series analysis of the physiotherapy workforce for each region of the country. The investigation of the nationwide distribution of physiotherapists in Brazil is necessary to further examine the relationship between supply and health needs and to support the attainment of the Universal Health Coverage. Thus, the objective of this study was to describe secular trends of the physiotherapists workforce-to-population ratio among the public and private health care sectors and across care levels (primary, secondary, tertiary) of the Unified Health System (*Sistema Único de Saúde*—SUS), by the five Brazilian geographical regions (North, Northeast, Central-west, Southeast, and South).

## Methods

A descriptive exploratory quantitative study based on secondary sources were obtained from the Brazilian National Registry of Health Care Facilities (*Cadastro Nacional de Estabelecimentos de Saúde*—CNES), the main national information system on health establishments, maintained and made publicly available by the Brazilian National Ministry of Health [20], available on the DATASUS website. Because this study used secondary data, it was exempt from the need for approval by the local Research Ethics Committee [21–23].

All data of the distribution of physiotherapists were collected monthly between August 2007 (when the Brazilian Classification of Occupations was updated in the DATASUS, equivalent to the physiotherapist at ISCO [24] code 2264) and September 2016 regarding facilities types, Brazilian regions and public and private sectors. Primary, secondary, and tertiary facilities were defined as follows: Primary Health Care (PHC) facilities—fitness centres, family health support clinics, general clinics (with various designations in Portuguese), clinics for indigenous peoples, mixed-use facilities, and mobile clinics; Secondary Health Care (SHC) facilities—transfusion medicine/hematology centres, psychosocial care centres, maternity centres, specialized outpatient clinics, physician offices, pharmacies, “polyclinics”, orthopaedic workshops, home care services, residential care clinics, and centres for diagnostic/therapeutic support; and Tertiary Health Care (THC) facilities—specialized hospitals, day hospitals, and general hospitals. The Brazilian regions are North, Northeast, Southeast, South and Central-west. The public sector is subsidized by the State and the payment in the private sector is exclusively an individual’s responsibility. Everyone in Brazil can use the public sector, while only people who can afford to pay for health care use the private sector.

We calculated coefficients for the number of physiotherapists per 1000 population in Brazil (nationwide and by region) to obtain the physiotherapy workforce-to-population ratio, in accordance with the guidelines established by the WHO in 2007 [25] (for example: 4782 physiotherapists of public PHC sector in August 2017 in Brazil/191741381 Brazilian population in August 2017 * 1.000), public and private health care facilities being evaluated separately. Data related to the population of Brazil for the 2007–2016 period were obtained from the Brazilian Institute of Geography and Statistics [26], which provides population estimates by performing geometric progression. The difference in means of the physiotherapy workforce-to-population ratio between the care level and between public and private sectors in Brazil was used one-way ANOVA and Bonferroni corrections were applied to the many levels, with a significance level of 0.05. The trends of the physiotherapy workforce-to-population ratio were visualized on maps generated through geoprocessing. Prais–Winsten linear regression models were used in order to estimate trends in the annual average of the physiotherapy workforce-to-population ratio, with a significance level of 0.05. The autocorrelation in the time series of the annual coefficients was checked by the Durbin–Watson test. Statistical procedures were performed in the Stata program, version 13.0 (StataCorp LP, College Station, TX, USA).

## Results

**Fig. 1:**
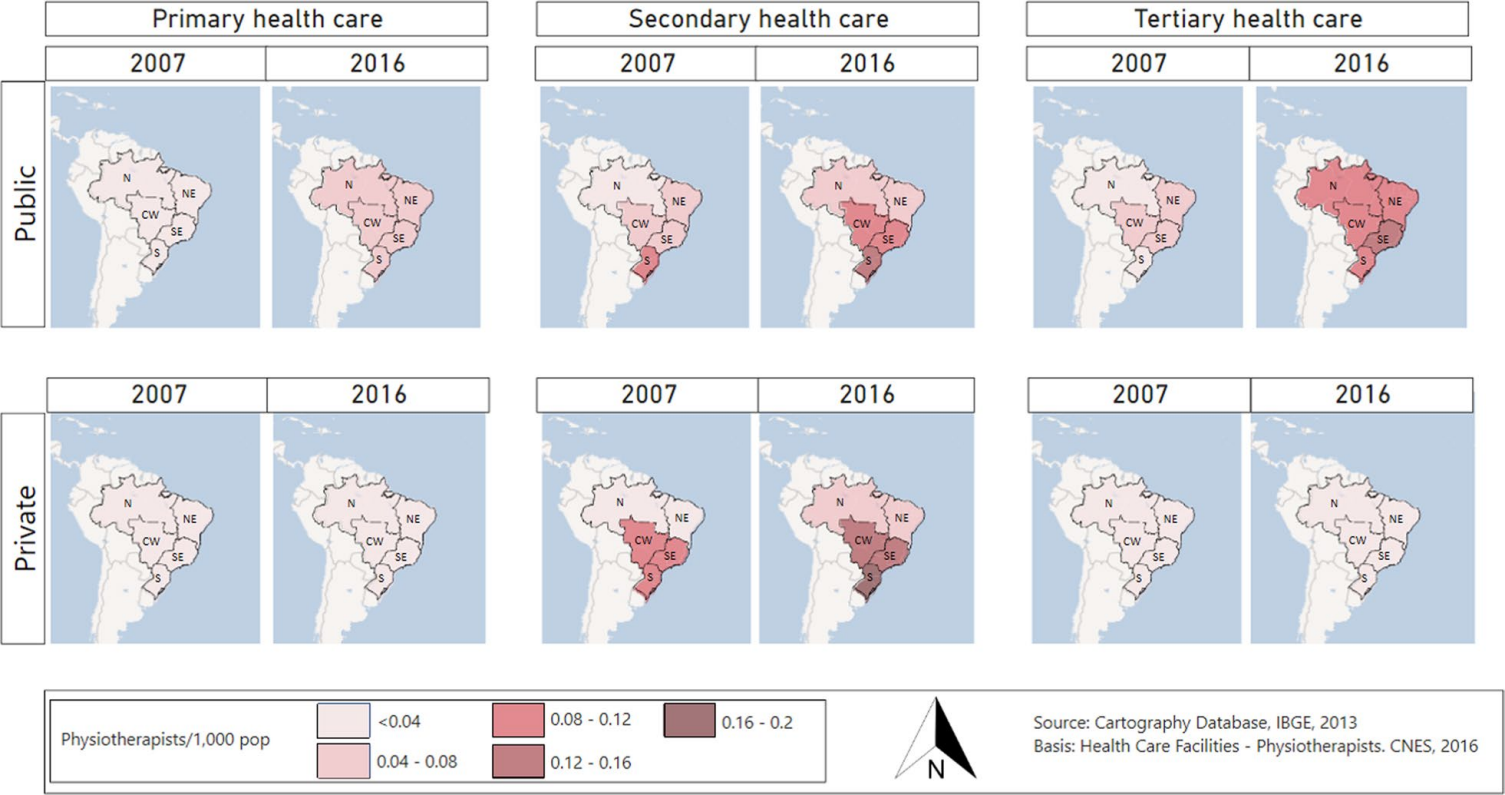
Physiotherapy workforce-to-population ratio, according to care level in public and private sectors, in the five geographic regions of Brazil (N: North, NE: Northeast, CE: Central-west, SE: Southeast, S: South). 2007 and 2016

**Fig. 2:**
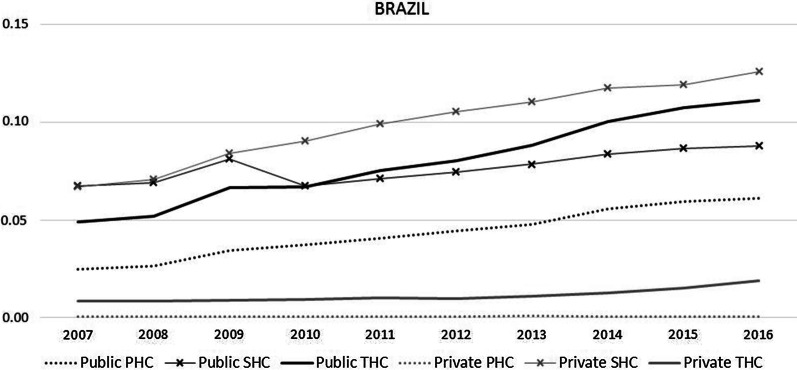
Physiotherapy workforce-to-population ratio, by sector and care levels, in Brazil. 2007–2016. PHC: primary health care; SHC: secondary health care; THC: tertiary health care

The physiotherapy workforce-to-population ratio in Brazil (public and private PHC, SHC and THC sectors) was 0.22 per 1000 population in August 2007 (42,164 physiotherapists; 191,741,381 population) and 0.41 per 1000 population in September 2016 (89,352 physiotherapists; 208,846,074 population), representing growth of 86%, with an increasing trend of 0.5% on an annual average (CI 95% 0.5–0.7%). There were clear increases in the physiotherapy workforce in all care levels of the public sector and in the private SHC sector. In 2016, the physiotherapy workforce-to-population ratio was higher for the private SHC level, followed by the public THC sector. There were regional differences, showing higher ratios mainly in the Southeast, South and Central-West regions (Fig. 1). After the workforce in public SHC dropped in 2009–2010, the public THC sector workforce surpassed the public SHC level (Fig. 2).

The public sector had the biggest physiotherapy workforce in the country in 2007 (65.1% for the three care levels *p* = *0.027*) and 2016 (64.4% for the three care levels *p* = *0.0209*). In 2016, the physiotherapy workforce-to-population ratio was bigger in the private SHC sector (0.1282 physiotherapist/1000 population), followed by the public THC sector (0.1145 physiotherapist/1000 population), the public SHC sector (0.0896 physiotherapist/1000 population) and the public PHC sector (0.0630 physiotherapist/1000 population). Among the care levels, PHC (*p* > *0.0001* for both years) and THC (*p* > *0.0001* for 2007 and *p* = *0.0420 for 2016*) levels had the smallest physiotherapy workforce-to-population ratio than SHC. In the private sector, workforce availability is highest at the SHC level, whereas in the public sector it is highest at the THC level. The workforce is larger in the public sector, *p* = *0.0274* for 2007 and *p* = *0.0209 for 2016* (Table 1).

**Table 1:** Physiotherapy workforce-to-population ratio, by sector and care level, in Brazil and regions. 2007 and 2016

*Region*	Year	Sector	Care level						Total		
			PHC		SHC		THC				
			Physiotherapist/1.000 population	%	Physiotherapist/1.000 population	%	Physiotherapist/1.000 population	%	Physiotherapist/1.000 population	%	
*Brazil*	2007	Public	0.0249	11.5	0.0673	31.1	0.0488	22.5	0.1411	65.1	***p* = *0.0274***^**a**^
		Private	0.0005	0.2	0.0669	30.9	0.0084	3.9	0.0757	34.9	
		Total	0.0254	11.7	0.1342	61.9	0.0572	26.4	0.2168	100	
			***p* < *0.0001***^**b**^								
					***p* < *0.0001***^**c**^						
			*p* = *0.0660*^d^								
	2016	Public	0.063	15.2	0.0896	21.6	0.1145	27.6	0.2671	64.4	***p* = *0.0209***^***a***^
		Private	0.0006	0.1	0.1282	30.9	0.0187	4.5	0.1474	35.6	
		Total	0.0636	15.3	0.2178	52.5	0.1331	32.1	0.4145	100	
			***p* < *0.0001***^**b**^								
					***p* = *0.0420***^**c**^						
			*p* = *0.1230*^**d**^								
*North*	2007	Public	0.0139	14.3	0.0309	32	0.0314	32.5	0.0762	78.8	***p* < *0.0001***^**a**^
		Private	0	0	0.0181	18.7	0.0025	2.5	0.0205	21.2	
		Total	0.0139	14.3	0.049	50.7	0.0339	35	0.0967	100	
			***p* = *0.0030***^**b**^								
					*p* = *0.3690*^**d**^						
			*p* = *0.1470*^d^								
	2016	Public	0.0452	19	0.0527	22.2	0.0842	35.5	0.1821	76.7	***p* < *0.0001***^***a***^
		Private	0.0003	0.1	0.0516	21.7	0.0034	1.4	0.0554	23.3	
		Total	0.0455	19.2	0.1043	43.9	0.0877	36.9	0.2375	100	
			*p* = *0.0580*^**a**^								
					*p* = *1.0000*^**c**^						
			*p* = *0.2630*^**d**^								
Northeast	2007	Public	0.0163	10.1	0.0581	36.2	0.0454	28.3	0.1198	74.6	***p* = *0.0003***^**a**^
		Private	0.0002	0.1	0.0363	22.6	0.0044	2.7	0.0408	25.4	
		Total	0.0164	10.2	0.0944	58.8	0.0498	31	0.1606	100	
			***p* < *0.0001***^**b**^								
					***p* = *0.0060***^**c**^						
			*p* = *0.0630*^d^								
	2016	Public	0.0788	22.2	0.0789	22.2	0,1126	31.7	0.2703	76.1	***p* < *0.0001***^**a**^
		Private	0.0008	0.2	0.0773	21.8	0,0065	1.8	0.0847	23.8	
		Total	0.0796	22.4	0.1562	44	0,1191	33.6	0.355	100	
			*p* = *0.1280*^a^								
					*p* = *0.9170*^c^						
			*p* = *0.8650*^d^								
Southeast	2007	Public	0.0326	12	0.079	29	0.0612	22.5	0.1728	63.5	***p* = *0.0394***^a^
		Private	0.0006	0.2	0.0845	31	0.0145	5.3	0.0996	36.5	
		Total	0.0332	12.2	0.1635	60	0.0756	27.8	0.2724	100	
			***p* < *0.0001***^**b**^								
					***p* < *0.0001***^**c**^						
			*p* = *0.0370*^d^								
	2016	Public	0.0593	12.3	0.0933	19.4	0.1314	27.3	0.2839	59.1	*p* = *0.1564*^a^
		Private	0.0005	0.1	0.1592	33.2	0.0366	7.6	0.1963	40.9	
		Total	0.0598	12.4	0.2525	52.6	0.1679	35	0.4803	100	
			***p* < *0.0001***^**b**^								
					*p* = *0.0600*^c^						
			***p* = *0.0150***^**d**^								
South	2007	Public	0.0343	11.8	0.0971	33.4	0.0389	13.4	0.1703	58.5	*p* = *0.3463*^a^
		Private	0.0011	0.4	0.1153	39.6	0.0042	1.5	0.1207	41.5	
		Total	0.0354	12.2	0.2124	73	0.0431	14.8	0.2909	100	
			***p* < *0.000*1**^***b***^								
					***p* < *0.0001***^**c**^						
			*p* = *1.0000*^d^								
	2016	Public	0.0635	12.7	0.1351	27.1	0.0948	19.1	0.2933	58.9	*p* = *0.2607*^**a**^
		Private	0.0005	0.1	0.1967	39.5	0.0072	1.5	0.2044	41.1	
		Total	0.0639	12.8	0.3318	66.6	0.1021	20.5	0.4978	100	
			***p* < *0.0001***^**b**^								
					***p* < *0.0001***^**c**^						
			*p* = *0.8020*^d^								
Central-west	2007	Public	0.0213	9.3	0.0539	23.6	0.0575	25.2	0.1327	58.1	*p* = *0.2379*^a^
		Private	0.0077	3.4	0.0802	35.1	0.0079	3.4	0.0957	41.9	
		Total	0.029	12.7	0.1341	58.7	0.0653	28.6	0.2285	100	
			***p* < *0.0001***^**b**^								
					***p* = *0.0010***^**c**^						
			*p* = *0.0830*^d^								
	2016	Public	0.057	14,4	0.0801	20.2	0.119	30.1	0,256	64,7	***p* = *0.0315***^**a**^
		Private	0.0101	2,5	0.1236	31.2	0.0062	1.6	0.1398	35,3	
		Total	0.067	16.9	0.2036	51.4	0.1252	31.6	0.3958	100	
			***p* = *0.0010***^**a**^								
					*p* = *0.0860*^c^						
			*p* = 0.3350^d^								

PHC, primary health care; SHC, secondary health care; THC, tertiary health care

^a^Anova between public and private

^b^Anova and Bonferroni correction between PHC and SHC level

^c^Anova and Bonferroni correction between SHC and THC level

^d^Anova and Bonferroni correction between PHC and THC level

Values of p showing significant differences are represented in bold

Complementing Table 1, sub-national regions show similar trends to the national estimates, with a difference for the North and Northeast regions. In 2007, North and Northeast region had the highest ratio in the public SHC sector (0.0309 and 0.0581 physiotherapist/1000 population, respectively) and the Southeast, South and Central-west regions in the private SHC sector (0.845, 0.1153 and 0.0802 physiotherapist/1000 population, respectively). In 2016, the private SHC sector in Brazil and its Southeast, South and Central-west regions had the potentialized rates (0.1592, 01,967 and 0.1236 physiotherapist/1000 population, respectively) as well as the public THC sector in North and Northeast (0.0842 and 0.1126 physiotherapist/1000 population, respectively).

For the trends of the annual average of the physiotherapists workforce-to-population ratio (Table 2), in Brazil from 2007 to 2016, the largest growth of the physiotherapy workforce-to-population ratio annual growth was in the public PHC sector (0.9% *p* > *0.001*), followed by the public and private THC sectors (0.8% *p* > *0.001*) and the SHC level, bigger in the private sector when compared to the public sector (0.6% *p* > *0.001* vs. 0.2% *p* = *0.004*). There were some regional trend differences in the annual average of physiotherapy workforce-to-population ratio to private PHC and THC sectors in North, Northeast, South and Central-West, but in general all five regions had greater growth of physiotherapy workforce-to-population ratio in the public PHC and THC sectors, followed by the private SHC sector.

**Table 2:** Trends of the annual average of the physiotherapists workforce-to-population ratio

Region	Sector	Trends of Physiotherapy Workforce %change annual average (95% CI) 2007–2016		
		PHC	SHC	THC
Brazil	Public	0.9 (0.7;1.1)^ab^ *p* < *0.001*	0.2 (0.1;0.4)^a^ *p* = *0.004*	0.8 (0.6;0.9)^ab^ *p* < *0.001*
	Private	0.2 (− 0.2;0.6) *p* = *0.362*	0.6 (0.5;0.7)^ab^ *p* < *0.001*	0.8 (0.5;1.0)^ab^ *p* < *0.001*
North	Public	1.0 (0.9;1.2)^ab^ *p* < *0.001*	0.4 (0.2;0.6)^a^ *p* < *0.001*	0.8 (0.7;0.9)^ab^ *p* < *0.001*
	Private	1.4 (0.6;2.1)^a^ *p* < *0.001*	0.9 (0.7;1.2)^a^ *p* < *0.001*	0.5 (0.2;0.9)^a^ *p* = *0.007*
Northeast	Public	1.5 (1.1;1.8)^ab^ *p* < 0.000	0.3 (0.1;0.4)^a^ *p* < 0.000	0.8 (0.7;0.9)^ab^ *p* < 0.000
	Private	1.5 (0.4;2.8)^a^ *p* = *0.007*	0.7 (0.5;0.8)^a^ *p* < *0.001*	0.3 (0.2;0.5)^a^ *p* < *0.001*
Southeast	Public	0.6 (0.4;0.7)^ab^ *p* < *0.001*	0.1 (−0.01;0.2) *p* = *0.117*	0.7 (0.5;0.9)^ab^ *p* = *0.014*
	Private	−0.2 (−0.4;−0.04) *p* < *0.001*	0.6 (0.4;0.8)^a^ *p* < *0.001*	0.9 (0.6;1.2)^a^ *p* < *0.001*
South	Public	0.6 (0.5;0.7)^ab^ *p* < 0.000	0.3 (0.1;0.4)a *p* < 0.000	0.8 (0.7;0.9)^ab^ *p* < 0.000
	Private	−0.8 (−1.6;−0.1)^c^ *p* = 0.019	0.5 (0.4;0.6)^a^ *p* < *0.001*	0.4 (0.2;0.6)^a^ *p* < *0.001*
Central-west	Public	0.9 (0.7;1.1)^ab^ *p* < *0.001*	0.3 (0.2;0.4)^a^ *p* < *0.001*	0.7 (0.6;0.7)^ab^ *p* < *0.001*
	Private	0.3 (0.1;0.5)^a^ *p* = *0.004*	0.4 (0.2;0.6)^a^ *p* < *0.0001*	−0.08 (−0.2;0.04) *p* < *0.221*

Brazil and regions, 2007–2016

PHC, primary health care; SHC, secondary health care; THC, tertiary health care

^a^Growth from 2007 to 2016

^b^Significant difference in relation to the care level

^c^Reduction from 2007 to 2016

## Discussion

As the workforce-to-population ratio approach is a critical component of monitoring and strengthening the performance of national health systems [18], this study was relevant to determine the availability of physiotherapists to the population of Brazil.

Regarding the *physiotherapy workforce in Brazil and in other countries*, the present study shows that the physiotherapy workforce-to-population ratio in Brazil (0.41 for 1000 people) is low in comparison with that estimated for other countries [13–16, 27]. According to Jesus et al. [13] Portugal had a ratio of 0.78 per 1000 people in 2014 and the United States a ratio of 0.65 per 1000 people. On the other hand, considering this study, Brazil has a greater workforce-to-population ratio when compared to Singapore (0.15) and Bangladesh (0.01). Also, Landry [14, 15] estimated the Canadian physiotherapy workforce-to-population ratio to be 0.5 in 2000. All these data may indicate that the physiotherapy workforce in Brazil is still insufficient to cover the population health needs.

However, our study used a method and a database that focused on physiotherapists that were available to provide health care in various facilities, while all compared studies used the number of registered professionals. Registered professionals may or may not be actually and directly providing health care, since they may be involved in management positions, research, education, or even unemployed. Our study analysed the availability of the physiotherapy workforce in various facilities, and also its distribution in the care levels and public and private sectors, bringing different perspectives to workforce data analysis and research.

An already known strategy used by many countries to increase the size of the workforce, is to increase the number of students. In Israel, the National Ministry of Health sought to control the size of the physician workforce increasing the number of medical students by 52%, resulting in a 32.5% increase in the size of the workforce [5]. But each country and region has an unique context, resulting in significant variability and requiring tailored solutions [13].

That is why many studies value the benchmark as an important tool to improve health care workforce analysis [4, 17, 27]. Brazil lags behind in the treatment of these data and the development of criteria to assess the demand for physiotherapy in Brazil as a whole and in its various regions, as well as the size of the physiotherapy workforce accordingly. Since an effective workforce is one of the global priorities highlighted by the WHO [1, 2], Brazil could find inspiration in other countries such as Australia and Israel [4, 5], as well as cities such as Hannover, Germany, and provinces as Saskatchewan, Canada [6, 16, 27]. Those places have all convened committees to study the markers of an effective workforce, to manage the demands related to access, and to create and implement public policies aimed at improving their health care systems.

We could also notice a plausible relation between the *distribution of the physiotherapy workforce in Brazil and the impact of public policies*. We found that the majority of physiotherapists in Brazil worked in the SHC sector, probably because the SHC sector was the “cradle” of physiotherapy in Brazil [19].

The trends related to growth of the physiotherapy workforce-to-population ratio in the SHC level were comparable between public and private sectors until 2009. In 2009 there was a decrease in the rate of physiotherapists in the public sector, whereas it kept growing in the private sector. This drop in the public SHC level could be explained by a partial targeting of professionals to the public PHC level, due to the creation and financing of the Family Health Support Center in 2008, that encouraged the position of the physiotherapist at the PHC level, expanding the physiotherapy workforce nationwide [28].

Another public policy that clearly affected the physiotherapy workforce distribution was a resolution launched in 2010 by the Brazilian Health Regulatory Agency (Anvisa) [29]. The resolution made it mandatory for every intensive care unit to have at least one physiotherapist team coordinator and at least one physiotherapist for every 10 beds [29], therefore increasing the physiotherapy workforce at the THC level. Three years after the enactment of the law, that workforce had grown by 0.8% in the public and private THC sectors.

With the objective of improving the Unified Health System, the structured implementation of the aforementioned public policies seems to be resulting in the inclusion of physiotherapy in various facilities and care levels, proving to be an important resource for modulating the healthcare workforce [4]. That is why, in order to adequately improve the access and distribution of the physiotherapy workforce in the five Brazilian regions and across care levels, it is indispensable to further investigate health needs with a local and community-centred approach.

While Brazilian public policies stipulate fixed and generalized incentives, without distinguishing the particular context, gaps and demands of the care levels and/or regions [5], the Israeli National Ministry of Health used the strategy of offering financial incentives according to their own scales, to bridge the gap between supply and demand [10]. Also, some countries like Israel and Australia have committees focused on understanding how to stimulate the health workforce towards the specializations and regions of greatest need [4, 5].

In Brazil we found a higher physiotherapy workforce mainly in the Southeast, South and Central-West regions, which can indicate bigger gaps in access to physiotherapy in other regions, which require further investigation. In a study conducted in Canada, Shah et al. [16] found that the physical and social barriers to physiotherapy healthcare access are greater in the smaller cities, where incomes are also lower. While the majority of physiotherapists work in urban areas, they found large gaps in access to physiotherapy in rural and remote areas. In order to improve the access to physiotherapy with greater equity, a similar analysis could contribute to planning a more appropriate physiotherapy assistance in the health system.

Nonetheless, there are some important issues to point out regarding the *access to physiotherapy and effective coverage in the Brazilian context*. Despite the concentration of physiotherapists being the highest at the SHC level, especially within the private sector (ratio of 0.13), that does not mean that the population has greater access to private SHC services of physiotherapy. According to the data from the 2013 Brazilian National Health Survey, only 27.9% of the Brazilian population has access to some type of private (i.e., only people who can afford to pay for health care) medical or dental insurance [30].

Overall, the imbalance found in the workforce distribution among the three levels of public and private health care and among the geographical regions may be a barrier to meeting the health needs and promoting access to health care services, especially for the poorest population [31]. Mcintyre et al. [32] discussed that access is more than simply having an opportunity to use the health care system. The end users of health care services should be well informed and must be encouraged to seek care and recognize health care as a right, as well as to understand that each individual plays an active role in requesting services and managing their own health care. However, promoting such empowerment is the responsibility of the healthcare system (to devise ways to overcome barriers) and of health professionals themselves (to be sources of information).

We hope that the data in this article will stimulate further studies on the relationship between the need for physiotherapy care and available physiotherapy workforce and their geographic distribution. It may also contribute to research regarding the rehabilitation workforce with the composition of other health professionals.

## Limitations of this study

The number of active physiotherapists might have been overestimated, because the CNES does not consider overlaps related to professionals with dual practice [17]. Since the CNES provides their data regarding the number of professionals registered in each facility and not about the amount of their working hours, an important limitation to this study methodology is the inability to present and calculate the professionals’ availability by full time equivalents. Other studies should consider and explore other methods of data collection that allow the adjustment of the professionals’ availability and working hours in each facility by full time equivalents.

Also, this study focused specifically on the physiotherapy workforce. Other studies comparing the workforce-to-population ratio, and its distribution and trends among other health professionals and health professionals overall would contribute for better understanding where the physiotherapy workforce stands in Brazil.

It is important to state the exploratory nature of the study. The adequate physiotherapy workforce-to-population ratio in order to assure effective health coverage is still unknown, therefore more investigation is needed to better understand and guarantee the access to physiotherapy, sufficiently meeting regional focused health needs for each level of care.

## Conclusion

There is a trend towards growth in the physiotherapy workforce in Brazil with differences between health care levels, and among public and private sectors. However, the physiotherapy workforce-to-population ratio appears to be still insufficient to meet the health needs of the population. The physiotherapy workforce-to-population ratio in Brazil is bigger in the private SCH sector, followed by the public THC, SHC and PHC sectors. The Brazilian regions followed similar patterns, with minor regional differences, mainly showing greater availability of the workforce in the Southeast, South and Central-West regions. Also, the physiotherapy workforce seems to have a close relationship with public policies related to human resources for health, therefore underscoring the importance of planning and regulation to meet the health needs of the population.

## Data Availability

The datasets used and/or analyzed during the current study are available from the corresponding author on reasonable request. However, the original data was obtained from the Brazilian National Registry of Health Care Facilities (*Cadastro Nacional de Estabelecimentos de Saúde*—CNES), available on the DATASUS website. Datasus. Brasil, Ministério da Saúde. http://datasus.saude.gov.br/sistemas-e-aplicativos/cadastros-nacionais/cnes. Accessed 11 Oct 2016.

## Abbreviations

CNES: National Registry of Health Care Facilities (*Cadastro* *Nacional* *de* *Estabelecimentos* *de* *Saúde*)
PHC: Primary Health Care
SHC: Secondary Health Care
THC: Tertiary Health Care
WHO: World Health Organization
